# Can Leprosy Reaction States Mimic Symptoms of Fibromyalgia? A Cross-Sectional Analytical Study

**DOI:** 10.3389/fmed.2022.870584

**Published:** 2022-04-25

**Authors:** Maria Stella Cochrane Feitosa, Gabriela Profírio Jardim Santos, Selma Regina Penha Silva Cerqueira, Gabriel Lima Rodrigues, Licia Maria Henrique da Mota, Ciro Martins Gomes

**Affiliations:** ^1^Programa de Pós-Graduação em Medicina Tropical, Faculdade de Medicina, Universidade de Brasília, Brasilia, Brazil; ^2^Programa de Pós-Graduação em Ciências Médicas, Faculdade de Medicina, Universidade de Brasília, Brasilia, Brazil; ^3^Programa de Pós-Graduação em Patologia Molecular, Faculdade de Medicina, Universidade de Brasília, Brasilia, Brazil

**Keywords:** leprosy, fibromyalgia, dermatology, rheumatology, diagnosis

## Abstract

Leprosy causes significant pain in affected patients, especially those experiencing reactional states. Fibromyalgia is characterized by widespread pain and is often accompanied by fatigue. Confusion between the clinical manifestations of fibromyalgia and those of leprosy reactions is possible at the primary care level, the first contact with the health system in most cases. We aimed to determine whether the presence of leprosy reactional states is related to the development of signs and symptoms included in the case definition of fibromyalgia and establish recommendations for obtaining the correct diagnosis. We performed a cross-sectional study in which the main independent variable was the presence of any leprosy reactional state and the primary dependent variable was the diagnosis of fibromyalgia according to the 2016 Revisions of the 2010/2011Fibromyalgia Provisional Criteria of the American College of Rheumatology. Forty-three patients were included in the study. Twenty-eight (65.12%) patients had a type I reactional state, only 1 (2.33%) had an isolated type II reactional state, and 5 (11.63%) had both type I and type II reactional states. Only 2 patients who suffered from cooccurring type I and II reactional states obtained sufficient scores for the diagnosis of fibromyalgia. Although diffuse pain was common in leprosy patients, none of the types of reactional states were associated with a higher frequency of criteria for fibromyalgia. We can conclude that a leprosy reactional state is probably not a risk factor for fibromyalgia but can act as a confounder, as tender points may be similar in both diagnoses. In patients diagnosed with fibromyalgia, leprosy must be considered in the differential diagnosis in endemic regions.

## Introduction

Leprosy is an infectious disease with chronic progression and intense immunological manifestations caused by *Mycobacterium leprae* and *Mycobacterium lepromatosis* (1). The disease mainly affects the skin and peripheral nerves. Early multidrug therapy is the best way to prevent transmission and morbidity (2–5). Leprosy reactions are acute immune-mediated episodes that are responsible for morbidity in most cases (6).

Fibromyalgia is present in 2-8% of the population (7, 8). The disease is characterized by widespread pain and is often accompanied by fatigue, memory problems, and sleep disturbances. After osteoarthritis, fibromyalgia is the second most common rheumatic disease (9). Its diagnostic criteria were originally published in 1990, and the symptoms emphasized were chronic generalized pain accompanied by tender points (7).

There seem to be no similarities between infectious diseases, such as leprosy, and chronic diseases, such as fibromyalgia. However, leprosy is a chronic disease that causes significant pain in affected patients and in advanced stages, especially in those experiencing reactional states (10). Chronic inflammation and the social stigma associated with leprosy also induce frequent constitutional symptoms (11). The importance of these two very different pathologies with many characteristics in common (12) will be addressed in this paper.

Patients who develop fibromyalgia usually have a lifelong history of chronic pain throughout the body (13, 14). They are more likely to have a history of headaches, dysmenorrhea, temporomandibular disorders, joint disorders, chronic fatigue, irritable bowel syndrome and other functional gastrointestinal disorders, interstitial cystitis, endometriosis, and other regional pain syndromes than those without fibromyalgia (7). Pain in the neck, back, shoulders, pelvic girdle, and hands is frequent; however, any part of the body can be affected. Leprosy can be associated with chronic pain due to immune-mediated neuropathy or direct action of the bacillus and thus can possibly be confused with fibromyalgia.

The main goal of this study was to determine whether the presence of leprosy reactional states is related to the development of signs and symptoms included in the case definition of fibromyalgia. Additionally, we aimed to monitor the frequency of leprosy symptoms that overlap with fibromyalgia and finally establish recommendations for the differential diagnoses of these two diseases to prevent any delay in diagnosis of leprosy.

## Methodology

### Study Design, Location and Circumstances of the Study

We performed a cross-sectional analytical study in which the main independent variable was the presence of any leprosy reactional state. The primary dependent variable was the diagnosis of fibromyalgia according to the 2016 Revisions to the 2010/2011 Fibromyalgia Diagnostic Criteria composed of the following diagnostic instruments (scales): the widespread pain index (WPI), symptom severity scale (SSS) and fibromyalgia severity (FS) score, which is the sum of the WPI and SSS (14). This is an updated version of the provisional criteria of the American College of Rheumatology (ACR) 2010 and the 2011 self-report modification for survey and clinical research and is widely used for fibromyalgia diagnosis. According to validation assessments, the criteria have an adequate sensitivity and specificity for the diagnosis and classification of fibromyalgia. The complete form can be found in Supplementary File 1 (14). Patients were consecutively recruited in 2020 and 2021 at the University Hospital of Brasília (HUB), which is a tertiary hospital for the care of patients with dermatological and rheumatological diseases.

### Inclusion Criteria

We included patients who received a formal diagnosis of leprosy according to the criteria of the World Health Organization (WHO), who were suspected of experiencing an active reactional state and who had not been diagnosed with fibromyalgia at the beginning of leprosy treatment according to the assessment instruments described elsewhere (1). The diagnosis of leprosy was always made by a dermatologist based on the criteria of the WHO (15), and at least one of the following findings was required for diagnosis: (1) skin lesion with altered sensitivity; (2) compatible peripheral neurological alterations, including asymmetric neural thickening, motor deformities, or hypoesthetic areas; (3) parasitological finding of *M. leprae* on smear examination or skin biopsy (performed for all patients in our center). The differential diagnosis of each included patient was systematically searched in our center by specialized clinical evaluation and complementary exams such as ultrasonography, electroneuromyography, and magnetic resonance imaging of the axial skeleton.

### Exclusion Criteria

We excluded patients from indigenous communities, and those who did not sign the consent form were excluded following local ethical restrictions.

#### Main Independent Variable Assessment

In all the included patients, the presence of a leprosy reactional state was evaluated by a dermatologist with a specialist title provided by the Brazilian Society of Dermatology. The classification of reactional states was also at the discretion of this expert and followed the WHO guidelines.

#### Primary Dependent Variable Assessment

The primary dependent variable was the diagnosis of fibromyalgia in the study population comprising only leprosy patients. Fibromyalgia was diagnosed according to the definition by Wolfe et al. (14). A patient fulfilled the diagnostic criteria for fibromyalgia if the following three conditions were met. (1) Part I of the WPI was equal to 7 and the parts IIa and IIb of the SSS were equal to 5, or the WPI ranged from 4 to 6, with an SSS score of 9. (2) Generalized pain, defined as pain in at least 4 of 5 regions, was present; pain in the jaw, chest, and abdomen was not included in the definition of generalized pain. (3) Symptoms were present for at least 3 months. A diagnosis of fibromyalgia was considered valid regardless of other diagnoses, except for leprosy in this specific case. A diagnosis of fibromyalgia does not exclude the presence of other clinically important diseases. Each variable and classification was analyzed for an association with the presence and type of any reactional state. We also considered the total sum of points (FS score) from the classification form for comparison.

#### Additional Variables

Additional factors were adjusted for when analyzing the above-described association, such as sex, age, personal history, family history, pathological history (mainly psychiatric illness), medication use, and social demographics. Leprosy classification was performed according to the Madrid classification, considering mainly the profile of skin lesions, to reduce the number of classification types and to improve the analytical approach (16). Leprosy classifications were as follows: indeterminate, tuberculoid, borderline, lepromatous, and pure neural leprosy. Joint and enthesis count, a clinical evaluation considered for the monitoring of disease activity of several rheumatic diseases, was also analyzed for an association with the presence of leprosy reactional states (17).

### Sample Size

Our target population was all patients with leprosy and with suspected active reactional states in the recruitment center.

### Statistical Analysis

After conducting interviews during clinical consultations, the data were anonymized to guarantee the confidentiality and identity of the patients. Initially, central tendency and dispersion values were calculated. The Analysis relied on a univariate approach followed by subgroup analysis, including the subanalyses of type I and II reaction states. Statistical analysis was performed with the “survminer” package of R version 0.4.9. (https://CRAN.R-project.org/package=survminer) (R Studio: Integrated development for R. R Studio, PBC, Boston, MA; URL http://www.rstudio.com/ by the free program R Core Team (2020); R: A language and environment for statistical computing. R Foundation for Statistical Computing, Vienna, Austria. URL https://www.R-project.org/). Statistical significance was defined as a *p*-value < 0.05 and a 95% confidence interval (CI).

### Ethical Aspects

The project was approved by the Ethics and Research Committee of the Faculty of Medicine of the University of Brasília (UnB), identification number CAAE: 07539519.2.0000.5558. All patients who agreed to participate in the study signed an informed consent form.

## Results

Forty-three patients were included in the study. The mean age of the participants was 46.81 years old (standard deviation = 13.39 years); 22 (51.16%) patients were female. The characteristics related to leprosy are shown in Table 1. Borderline leprosy was the most frequent form, affecting 30 (69.77%) patients. Eleven patients were using alternative treatment. Twenty-eight (65.12%) patients had a type I reactional state when evaluated, only 1 (2.33%) had an isolated type II reactional state and 5 (11.63%) had both type I and type II reactional states. The medications used to treat reactional episodes are shown in Table 1. None of the included patients presented associated rheumatologic or neurologic conditions that could explain diffuse pain episodes.

**Table 1:** Clinical variables related to leprosy classification and treatment.

**Variable**	**Frequency (*n*)(%)**
**Leprosy classification**	
Borderline	30(69.77%)
Lepromatous	7(16.28)
Pure neural leprosy	6(13.95%)
**Treatment**	
Standard multidrug therapy	32(74.42%)
Alternative treatment	11(25.58%)
**Reactional state**	
Type I	28(65.12%)
Type II	1(2.33%)
Types I and II	5(11.63%)
No reactional state	9(20.93%)
**Medications for reactional states**	
Prednisone	30(69.77%)
Thalidomide	4(9.30%)
Pentoxifylline	2(4.65%)

*n, number of patients*.

### Analysis of the Primary Dependent Variable

In the present study, only two patients who were simultaneously affected by types I and II reactions obtained sufficient scores for the diagnosis of fibromyalgia according to the adopted criteria. None of the types of reactional states were associated with a higher frequency of criteria for fibromyalgia. Multivariate analysis was not performed due to the small number of patients with a diagnosis of fibromyalgia, the primary dependent variable.

Although only two patients had a complete diagnostic criterion of fibromyalgia, only two patients had no points scored in the WPI and SSS scores. All remaining patients had at least one unspecific sign or symptom that could be found in patients with fibromyalgia. Among all the patients, the median part I WPI was 1.00, with an interquartile range of 2.00. The median (interquartile range) numbers of part IIa and part IIb WPIs were 2.00 (6.00) and 1.00 (1.50), respectively. The median total score (FS score) in the studied population was 4.00, with an interquartile range of 8.00. The median joint count was 0 in the total population, with an interquartile range of 3.00. The median and interquartile range of the enthesis count was 0. The analysis of the scores obtained from the assessment tool is shown in Table 2.

**Table 2:** Comparison between the evaluation of the Widespread Pain Index (WPI) (part I), the Symptom Severity Score (SSS) (parts IIa and IIb) and the fibromyalgia severity (FS) score with the presence of leprosy reactional states.

	**Type I reaction Median (interquartile range)**		
**Criteria**	**Yes**	**No**	***p*-value**
WPI part I	1.00(2.00)	0.50(1.75)	0.690
SSS part IIa	2.00(6.00)	2.00(5.00)	0.596
SSS part IIb	1.00(1.00)	2.00(1.00)	0.013
FS score	4.00(8.00)	3.50(8.75)	0.839
Joint count	0(3.00)	0(2.50)	0.766
Enthesis count	0(0)	0(0)	0.816
	**Type II reaction Median (interquartile range)**		
**Criteria**	**Yes**	**No**	***p*** **-value**
WPI part I	0(0)	1(2.75)	0.028
SSS part IIa	2.00(1.00)	3.00(6.00)	0.485
SSS part IIb	1.00(1.50)	1.00(1.00)	0.895
FS score	3.50(4.00)	4.0(8.00)	0.408
Joint count	0(0)	0.50(3.00)	0.149
Enthesis count	0(0)	0(0)	0.878
	**Simultaneous type I and II reactions Median (interquartile range)**		
**Criteria**	**Yes**	**No**	***p*** **-value**
WPI part I	1.00(2.00)	0(1.00)	0.493
SSS part IIa	2.50(6.00)	1.00(5.00)	0.258
SSS part IIb	1.00(1.00)	2.00(1.00)	0.033
FS score	5.00(8.00)	3.00(8.00)	0.539
Joint count	0.50(3.00)	0(1.00)	0.526
Enthesis count	0(0)	0(0)	0.968

*WPI, Widespread Pain Index; SSS, symptom severity scale; FS, fibromyalgia severity*.

## Discussion

Leprosy is essentially a disease of the peripheral nerves and is the most frequent cause of treatable peripheral neuropathy in endemic countries (6, 18, 19). In the initial phase of the disease, invasion of the dermal and superficial peripheral nerves occurs, resulting in reduced thermal, pain and tactile sensitivity, often preceded by autonomic changes, such as lack of sweating and hair loss. Subsequently, neuropathic pain and deformity can occur as a result of an immune-mediated reaction targeting neural structures (5). Although the leprosy case detection rate is considered very low in developed countries such as the United Kingdom (2 new cases in 2019) and the United States of America (no new cases in 2019), in tropical countries and in countries in development such as Brazil (27.863 new cases in 2019) and India (114.451 new cases in 2019) this is a relevant public health problem (20). In addition, crescent migration waves show that leprosy can be a possible reality in any world region.

In the clinical evaluation of a leprosy patient, a history of pain and/or paraesthesia in the areas corresponding to the affected nerves, as well as the sensation of numbness in extremities or other specific areas of the skin, are important symptoms (21, 22). In addition, acute manifestations induced by reactional states may develop, resulting in progression to chronic neuropathy with persistent pain (23). When chronic neuropathy persists, complications such as osteomyelitis and plantar perforating disease can be aggravating factors. The present study, which was performed in a tertiary hospital located in a leprosy-endemic country, demonstrated that most patients were affected by type I leprosy reactions (33 patients), and only 6 had type II leprosy reactions. A minority of the evaluated patients did not develop a reactional state.

Fibromyalgia syndrome is characterized by pain throughout the body, especially in the musculature (24, 25). Symptoms such as fatigue, non-restorative sleep, memory and attention disorders, anxiety, depression and intestinal disorders are associated with fibromyalgia pain. Since pain is the primary manifestation in fibromyalgia, fibromyalgia can be confused with leprosy, which is also characterized by pain as an initial symptom. Although most leprosy patients included in this study were experiencing reactional states, only two patients had a clinical picture compatible with fibromyalgia. However, most leprosy patients reported multiple points of pain according to the tool, showing that confusion between these 2 diseases may occur during non-specialized evaluations.

Leprosy and its reactions can mimic many dermatological, rheumatological, and neurological diseases (26–28). The most characteristic sign of leprosy is skin hypoesthesia or anesthesia, while peripheral neuropathy is also an important manifestation of this infectious disease. In some cases, no skin changes are recognized, and these cases are considered pure neural leprosy. The real incidence of leprosy without skin manifestations is unknown, but this type of leprosy is probably underdiagnosed. There are many rheumatological and neurological diseases that induce changes in peripheral sensitivity. The data from the present study show some similarities between leprosy reactions and fibromyalgia that can be confused especially by poorly trained providers, but the applied tool seems to be adequate for the differentiation of the two conditions. This means that both conditions are possibly confused only in cases in which clinical examination is not proper. Specific educational interventions must be directed to healthcare providers at the primary care level and in underdeveloped areas where leprosy is endemic.

Fibromyalgia is a disease with a certain degree of difficulty in definitive diagnosis, as no definitive laboratory standard exists. Our results clearly showed that confusion between the clinical manifestations of fibromyalgia and those of leprosy reactions is possible (12). Fibromyalgia is a common disease with a prevalence of more than 20% prevalence in rheumatology clinics, even in regions where leprosy is not endemic; this suggests that relevant epidemiological information, especially a history of household contact with a leprosy patients or migration from an endemic country, must be collected and considered (8). Fibromyalgia is characterized by tendon and muscle pain, adynamia, paraesthesia, and non-specific symptoms, but it does not manifest as neuropathy. Changes in the sensitivity of peripheral nerves should be investigated in patients with suspected fibromyalgia, especially in regions where leprosy is endemic. This is the most important clue for differential diagnosis (12). Healthcare providers must be aware that both diseases can occur concurrently, with overlapping or mimicking signs and symptoms.

Although our entire target population was evaluated, the size of our study population is a probable limitation for the assessment of causal association. Future longitudinal studies may use the present data for the calculation of associations. Additionally, data were collected with the use of a questionnaire; therefore, a certain degree of subjectivity is expected. This is a constant limitation for the evaluation of diseases that have no laboratory or gold standard diagnostic criteria (3, 5).

The present study allows a better understanding of the relationship between leprosy and fibromyalgia and thus contributes to a better understanding of the control of chronic pain in leprosy; this study also contributes to the analysis of the differential diagnosis of these two diseases. Rheumatologists and clinicians must be careful when evaluating and diagnosing fibromyalgia, as it is important to consider the possibility of a diagnosis of leprosy, which can be a confounder, in patients in endemic countries.

In conclusion, a leprosy reactional state is not a risk factor for fibromyalgia but can act as a confounder. Tender points are common in both diagnoses. Several chronic pain points were present in patients with leprosy and leprosy reactions, but only 2 patients met the diagnostic criteria for fibromyalgia according to the tool. New prospective studies must be conducted to better elucidate this issue. Finally, patients diagnosed with fibromyalgia must be actively evaluated for leprosy during the differential diagnosis in regions where leprosy is endemic.

## Data Availability Statement

The raw data supporting the conclusions of this article will be made available by the authors, without undue reservation.

## Ethics Statement

The studies involving human participants were reviewed and approved by Faculdade de Medicina da Universidade de Brasília. All patients who agreed to participate in the study signed an informed consent form.

## Author Contributions

MF and SC: investigation. GS, MF, CG, and LM: formal analysis. GS, MF, LM, CG, and SC: resources. GR and CG: visualization, writing—original draft, and writing—review and editing. CG: conceptualization, data curation, funding acquisition, methodology, project administration, software, supervision, and validation. All authors contributed to the article and approved the submitted version.

## Funding

This study was financed in part by Fundo de Apoio à Dermatologia (FUNADERM)—Sociedade Brasilleira de Dermatologia (SBD).

## Conflict of Interest

The authors declare that the research was conducted in the absence of any commercial or financial relationships that could be construed as a potential conflict of interest.

## Publisher's Note

All claims expressed in this article are solely those of the authors and do not necessarily represent those of their affiliated organizations, or those of the publisher, the editors and the reviewers. Any product that may be evaluated in this article, or claim that may be made by its manufacturer, is not guaranteed or endorsed by the publisher.
