# Supplementary material for: Can Leprosy Reaction States Mimic Symptoms of Fibromyalgia? A Cross-Sectional Analytical Study
Source: Front Med (Lausanne). 2022 Apr 25;9:870584. doi: 10.3389/fmed.2022.870584 (PMC9082667; doi:10.3389/fmed.2022.870584)
Supplement: Supplementary file 1 [file Data_Sheet_1.docx]

**Can leprosy reaction states mimic symptoms of fibromyalgia? a cross-sectional analytical study**

Fibromyalgia criteria—2016 revision according to Wolfe F, Clauw DJ, Fitzcharles M-A, Goldenberg DL, Häuser W, Katz RL, Mease PJ, Russell AS, Russell IJ, Walitt B. 2016 Revisions to the 2010/2011 fibromyalgia diagnostic criteria. Semin Arthritis Rheum (2016) 46:319–329. doi:10.1016/j.semarthrit.2016.08.012

**Fibromyalgia case definition**

**Criteria**

**A patient satisfies modified 2016 fibromyalgia criteria if the following 3 conditions are met:**

(1) Widespread pain index (WPI) ≥ 7 and symptom severity scale (SSS) score ≥ 5 OR WPI of 4–6 and SSS score ≥ 9.

(2) Generalized pain, defined as pain in at least 4 of 5 regions, must be present. Jaw, chest, and abdominal pain are not included in generalized pain definition.

(3) Symptoms have been generally present for at least 3 months.

(4) A diagnosis of fibromyalgia is valid irrespective of other diagnoses. A diagnosis of fibromyalgia does not exclude the presence of other clinically important illnesses.

**Ascertainment**

**(1) WPI:** note the number of areas in which the patient has had pain over the last week. In how many areas has the patient had pain? Score will be between 0 and 19

Left upper region (Region 1)

Jaw, left

Shoulder girdle, left

Upper arm, left

Lower arm, left

Right upper region (Region 2)

Jaw, right

Shoulder girdle, right

Upper arm, right

Lower arm, right

Left lower region (Region 3)

Hip (buttock, trochanter), left

Upper leg, left

Lower leg, left

Right lower region (Regin 4)

Hip (buttock, trochanter), right

Upper leg, right

Lower leg, right

Axial region (Region 5)

Neck

Upper back

Lower back

Chest

Abdomen

**2) Symptom severity scale (SSS) score**

Fatigue

Waking unrefreshed

Cognitive symptoms

For the each of the 3 symptoms above, indicate the level of severity over the past week using the following scale:

0 = No problem

1 = Slight or mild problems, generally mild or intermittent

2 = Moderate, considerable problems, often present and/or at a moderate level

3 = Severe: pervasive, continuous, life-disturbing problems

**The symptom severity scale (SSS) score:** is the sum of the severity scores of the 3 symptoms (fatigue, waking unrefreshed, and cognitive symptoms) (0–9) plus the sum (0–3) of the number of the following symptoms the patient has been bothered by that occurred during the previous 6 months:

(1) Headaches (0–1)

(2) Pain or cramps in lower abdomen (0–1)

(3) And depression (0–1)

The final symptom severity score is between 0 and 12

**The fibromyalgia severity (FS) scale** is the sum of the WPI and SSS
